# *Corynebacterium striatum* thrombophlebitis: a nosocomial multidrug-resistant disease?

**DOI:** 10.1099/acmi.0.000307

**Published:** 2021-12-17

**Authors:** Julie Tang, Dimitri Kornblum, Nagisa Godefroy, Gentiane Monsel, Jérome Robert, Eric Caumes, Valérie Pourcher, Elise Klement-Frutos

**Affiliations:** ^1^​ Sorbonne Université, Assistance Publique-Hôpitaux de Paris, Service des Maladies Infectieuses et Tropicales, Hôpital Pitié-Salpêtrière, 75013-Paris, France; ^2^​ Sorbonne Université, Assistance Publique-Hôpitaux de Paris, Service de Bactériologie et d’Hygiène hospitalière, Hôpital Pitié-Salpêtrière, 75013-Paris, France

**Keywords:** *Corynebacterium*, septic thrombophlebitis, multidrug resistance

## Abstract

**Introduction:**

*
Corynebacterium striatum* is a non-*Diphteriae* commensal bacterium with a wide range of pathogenicity. The identification of multidrug-resistant (MDR) *C. striatum* is concerning because drug susceptibility testing is not usually performed in microbiology laboratories. There is no consensus yet on the treatment of septic thrombophlebitis in this situation.

**Case report:**

We report here the first case of a quinquagenarian patient with a history of AIDS and fungic endocarditis, who was diagnosed with a nosocomial thrombophlebitis in the right jugular vein caused by *C. striatum *. Bitherapy with daptomycin for 12 days and linezolid for 23 days was combined with a therapeutic anticoagulant. The follow-up included weekly cervical ultrasound controls. The efficiency of the treatment and the stability of the lesions allowed us to alleviate the medication with a prophylactic dose of anticoagulant. The patient was discharged from hospital and showed no signs of recurrence after 12 months.

**Conclusion:**

The lack of consensus relative to the management of septic thrombophlebitis precludes the validation of a specific treatment for the condition. Our results suggest that a combination that includes removal of the medical device is needed. A total of 6 weeks of antibiotherapy should be applied, starting with 2 weeks of vancomycin or a combination of antibiotitherapy with daptomycin in order to reduce the bacterial load and avoid resistance. Six weeks of anticoagulation therapy is effective.

## Introduction

*Corynebacterium striatum* is a Gram-positive rod-shaped bacterium that is a commensal of the skin and respiratory tract with a human pathogenesis that is now recognized [1–3]. Although endocarditis is the most frequent clinical form, other severe infections have been reported, such as pleuropulmonary infections, breast and pancreatic abscesses, peritonitis, septic arthritis, osteomyelitis, ventricular device infections and even urinary tract infections [1, 4–11]. *C. striatum* is now increasingly implicated in nosocomial sepsis, frequently associated with medical devices [3, 9]. Furthermore, the development of antibiotic resistance among *C. striatum *, especially to daptomycin, is alarming [1–13].

We hereby present a rare clinical case of nosocomial *C. striatum* septic thrombophlebitis. While the standard care for septic thrombophlebitis is still controversial, the antibiotic treatment to use when *C. striatum* is the pathogen is even less clear [14–16]. By presenting this case and an updated literature review, we aim to highlight the increase of multidrug-resistant (MDR) *C. striatum* frequency and pathogenicity, and discuss its management.

## Case report

A quinquagenarian patient with a 10-year history of AIDS was hospitalized in September 2019 for mitral infectious endocarditis (IE) caused by *Candida albicans*. The patient also had a previous history of peritonitis and intestinal occlusion leading to a colostomy. He had a chronic arterial ulcer on the right lower leg, but the local cultures realized in the past and during the hospitalization remained inconclusive. IE vascular complications comprised a left arm thrombosis treated using an arteriotomy and embolectomy, a splenic embolus and multiple cerebral ischemias with a favourable evolution. The patient underwent surgical replacement of the mitral valve by a bioprosthesis on day 2 and empirical antimicrobial therapy combining vancomycin, ceftriaxone and caspofungin. Subsequent to the microbiological identification of *C. albicans*, antibiotics were discontinued and the antifungal treatment was switched to fluconazole, with an 800 mg bolus followed by a dose adapted to his renal function for a total of 6 weeks. The antiretroviral treatment was reintroduced as the HIV plasma viral load was above 6 log and the CD4 count was 37 mm^−3^.

Two weeks later, on 24 October 2019, the patient presented a 40 °C fever with an inflammatory syndrome (C-reactive protein at 138 mg l^−1^) and a hyperleukocytemia (neutrophil polynuclear cells at 9 g l^−1^), which led to the removal of his right jugular catheter and the introduction of empirical treatment with piperacillin/tazobactam (4 g/6 h) and daptomycin (10 mg/kg/day).

On 25 October 2019, the blood cultures (BACT/ALERT technique) and culture of the removed catheter (quantitative Brun-Buisson technique [17]) revealed the presence of *C. striatum *. One blood culture of two bottles drawn from the catheter and one blood culture of two peripheral bottles drawn simultaneously showed the presence of *C. striatum *. The first one was positive 4.5 h earlier than the second, suggesting a central line-associated bloodstream infection.

Drug susceptibility tests were performed using the disc diffusion method and interpreted according to the national recommendations (CA-SFM 2018). The strain was resistant to β-lactam, clindamycin, cotrimoxazole and gentamicin, and susceptible to vancomycin, rifampicin, linezolid and daptomycin, with an MIC of 0.047 mg l^−1^. The MIC for daptomycin was determined using the Etest strip method (BioMérieux).

**Fig. 1.**
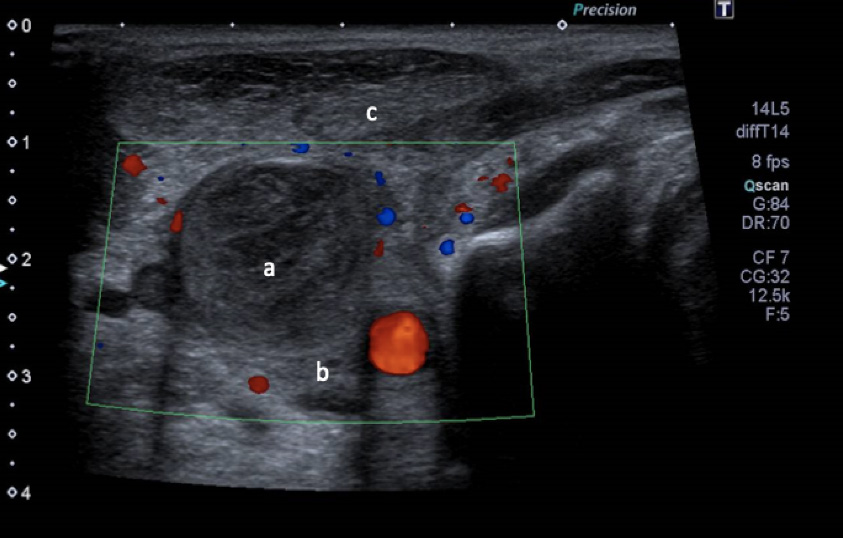
Ultrasound Doppler of the right cervical region performed on 6 November 2019: signs of an enlarged right internal jugular vein with heterogeneous echogenic material (**a**) over its entire height and taken over by collaterals, which led to the diagnosis of a recent thrombosis of the right internal jugular vein; signs of an infiltration and thickening of soft cervical tissue (**b**); numerous right cervical infracentimetric lymph nodes; no abscess of the soft parts of the sterno-cleido-mastoid muscle (**c**) was observed.

The *C. striatum* septicaemia was initially treated using antibiogram-adapted vancomycin monotherapy at 30 mg/kg/day (continuous intravenous), with a vancomycin concentration of 29.4 mg l^−1^ at day 2, and negative blood cultures from 28 October 2019. Vancomycin was stopped on 3 November 2019 due to an acute kidney injury with a creatinine level of 6 mg dl^−1^ and replaced by intravenous linezolid 600 mg day^−1^ adapted to the kidney function. The vancomycin concentration was 47 mg l^−1^ on 2 November 2019 and 30 mg l^−1^ on 4 November 2019. On 5 November 2019, the patient complained of a febrile painful dysphagia attributed to a right cervical tumefaction palpable on the site of the previous catheter. A thoraco-abdominal CT scan, performed on 5 November 2019 without injection of contrasting agent because of renal failure, was inconclusive. The C-reactive protein level was 234 mg l^−1^, harvested blood cultures showed no presence of *C. striatum* and a cardiac echography excluded an infectious endocarditis. However, on 6 November 2019 a cervical echography showed a recent large thrombosis of the right jugular vein (Fig. 1). Daptomycin (10 mg/kg/day) was added to linezolid, which was then given orally, and therapeutic anticoagulation using calciparine 250 IU kg^−1^ was started.

After 3 days of antibiotic combination, the fever, the volume of the cervical swelling and some biological inflammatory indicators started to decrease. Daptomycin was discontinued after 12 days. The cervical ultrasound Doppler performed at weeks 1 and 2 revealed a persistent thrombosis up to 50 mm, but no further extension. Anticoagulation treatment was reduced in favour of a prophylactic treatment on 27 November 2019 in order to minimize the adverse effects as the thrombosis had evolved to chronicity. The weekly blood cultures for 4 weeks remained negative, and the C-reactive protein decreased to 10 mg ml^−1^. Linezolid was stopped after 23 days. One month post-treatment the patient had fully recovered and was referred to a rehabilitation centre. He had not relapsed at 12 months.

## Discussion

*C. striatum* is increasingly described in severe infections and is now the most frequently detected non-diphtheriae *Corynebacterium* in nosocomial infections, yet the understanding of its pathogenicity remains fragmented [16]. Its involvement in another case of septic thrombophlebitis in an immunosuppressed patient reported by Elfenbein *et al*. was declared *nomen dubium* due to wrong strains of identification of this underspecies. Hence, misidentifications of *C. striatum* have been described, which raises some uncertainty regarding the validity of the previous report [3, 18, 19]. According to the literature, a catheter-related septic thrombophlebitis should be suspected after 72 h of antibiotherapy without clinical improvement, and confirmed by radiographic imaging and blood cultures [20]. Therapeutic anticoagulation had previously been suggested for septic deep thrombosis, but no consensus has been established to date [13, 21].

This study has some limitations. The bacterial identification was only performed using BACT/ALERT. Indeed, as the phenotypic concordance seemed effective, it is not common to use genotypic typing to compare strains among the same patient from samples that have been drawn simultaneously and are positive with such an uncommon pathogen. Moreover, we suggest an efficient therapy based on one case and the literature (a few cases).

We would like to emphasize the importance of combining the removal of the medical device with an intravenous, proven, effective antibiotic treatment and therapeutic anticoagulation [3, 10, 16]. The most used antibiotic is vancomycin, which is considered to be the reference treatment of *Corynebacteria* bacteriaemia in the absence of resistance, but its use is limited by renal toxicity. In the report case, we observed the occurrence of the clinical signs of thrombosis despite 10 days of active antibiotic treatment. This could be caused by the ability of the bacteria to produce biofilms, although it has not been tested [3, 14]. Since a serious concern was raised about daptomycin resistance, we chose to treat the patient with a combination of daptomycin 10 mg/kg/day and linezolid [3, 22, 23]. We also performed weekly blood cultures during treatment and after it had ended. Although in most studies follow-up was interrupted after 3–6 months, a case report of recurrence after 20 months may suggest that a longer follow-up is required in the case of IE [15]. In this report of *C. striatum* septic thrombophlebitis, the removal of the device followed by a combination of two antibiotics proven to be active for 4 weeks after the last positive blood culture and a therapeutic anticoagulant for 6 weeks allowed the patient to recover. Unlike in previous studies, a Doppler ultrasound was realized after the first week of anticoagulation therapy, and showed the stability of the thrombosis, contrasting with the clinical and biologicla improvement [16].

*C. striatum,* a common component of the skin microbiota, can infect medical devices such as catheters directly, or, as some authors have suggested, secondarily from a chronic skin wound, which may have been the case in this clinical report [3, 24]. To the best of our knowledge, no association with HIV infection has been reported before.

## Conclusion

We report here the first deep septic thrombophlebitis and bacteraemia superimposed by multidrug-resistant *Corynebacterium striatum* in France. The increasing uses of invasive devices, aggressive antimicrobial therapies and immunosuppressive treatments can only lead to these types of nosocomial infections [3, 25]. In the absence of a consensus on the general recommendations to treat *C. striatum* septic deep thrombophlebitis, we suggest that treatment should associate the removal of the medical device with at least 4–6 weeks of susceptibility-directed antibiotic treatment, starting with vancomycin, or daptomycin used in biantibiotherapy, and 6 weeks of anticoagulation therapy. If confronted by an MDR corynebacterium, new antibiotics such as dalbavancin could be considered, but still need to be clinically evaluated [26, 27]. Moreover, the risk of recurrence of this infection makes it important to closely monitor and reconsider the duration of the treatment, especially in cases of infectious endocarditis and immunocompromised patients.
